# Comparative Study between Perfusion Changes and Positive Findings on Coronary Flow Reserve

**DOI:** 10.5935/abc.20160184

**Published:** 2017-01

**Authors:** Costantino Roberto Frack Costantini, Jose Antonio Ramires, Costantino Ortiz Costantini, Marcos Antonio Denk, Sergio Gustavo Tarbine, Marcelo de Freitas Santos, Daniel Aníbal Zanuttini, Carmen Weigert Silveira, Admar Moraes de Souza, Rafael Michel de Macedo

**Affiliations:** 1 Hospital Cardiológico Costantini, Curitiba, PR - Brazil; 2 Instituto do Coração (InCor) - Faculdade de Medicina da Universidade de São Paulo, São Paulo, SP - Brazil

**Keywords:** Coronary Artery Disease / mortality, Percutaneous Coronary Intervention, Myocardial Ischemia, Fractional Flow Reserve, Myocardial / physiology

## Abstract

**Background:**

Functional assessment of coronary artery obstruction is used in cardiology practice to correlate anatomic obstructions with flow decrease. Among such assessments, the study of the coronary fractional flow reserve (FFR) has become the most widely used.

**Objective:**

To evaluate the correlation between FFR and findings of ischemia obtained by noninvasive methods including stress echocardiography and nuclear medicine and the presence of critical coronary artery obstruction.

**Methods:**

Retrospective study of cases treated with systematized and standardized procedures for coronary disease between March 2011 and August 2014. We included 96 patients with 107 critical coronary obstructions (> 50% in the coronary trunk and/or ≥ 70% in other segments) estimated by quantitative coronary angiography (QCA) and intracoronary ultrasound (ICUS). All cases presented ischemia in one of the noninvasive studies.

**Results:**

All 96 patients presented ischemia (100%) in one of the functional tests. On FFR study with adenosine 140 g/kg/min, 52% of the cases had values ≤ 0.80. On correlation analysis for FFR ≤ 0.80, the evaluation of sensitivity, specificity, positive and negative predictive values, accuracy, and ROC curve in relation to the stenosis degree and length, and presence of ischemia, no significant values or strong correlation were observed.

**Conclusion:**

Coronary FFR using a cut-off value of 0.80 showed no correlation with noninvasive ischemia tests in patients with severe coronary artery obstructions on QCA and ICUS.

## Introduction

Coronary artery disease (CAD) is considered the most common cause of death due to cardiovascular diseases (CVD) in Brazil and worldwide. Nonetheless, the number of individuals aged more than 60 years who survive a first event increases at each year, a fact that is attributed to technological advancements in diagnostic methods and treatment techniques over the past 30 years.^1-3^

International guidelines recommend a combination of functional and anatomical assessments to define the ideal treatment strategy for CAD.^4,5^ However, some studies^6-10^ aiming at complete lesion revascularization, have proposed treatment of lesions with a ≤ 50% stenosis diameter with percutaneous coronary intervention (PCI), prioritizing the anatomical findings independent of their functional repercussions (assessed by noninvasive methods).

The DEFER study showed that it is safe to defer treatment of functionally nonsignificant coronary lesions.^11^ More recently, the FAME study showed that in the presence of multivessel disease, treatment of epicardial lesions guided by fractional flow reserve (FFR) is associated with a reduction in ischemic complications when compared with treatment guided by angiography.^12^

Based on these findings, FFR measurement has become routine in guiding clinical decision making in CAD treatment. However, both the technique and its cut-off value of 0.80 have not been tested in some specific situations including severe coronary artery obstructions (the initial results involved minor and moderate lesions). Therefore, to evaluate the impact of FFR measurement on severe lesions with ischemia previously detected by noninvasive functional tests will be of great importance, as the decision to treat or not to treat these lesions may be substantiated by the results of the FFR study.

Thus, the objective of this study was to correlate the FFR results, using a cut-off value of 0.80, with the presence of ischemia, detected by noninvasive tests including stress echocardiography or nuclear medicine, in patients with severe coronary obstruction assessed by cineangiography and intracoronary ultrasound (ICUS).

## Methods

### Type of study

We conducted a retrospective study of cases treated with systematized and standardized procedures for coronary disease between March 2011 and August 2014 at the *Hospital Cardiológico Costantini (HCC)* in Curitiba.

### Studied population

We screened 264 patients with suspected CAD who had undergone noninvasive functional tests, pharmacological stress echocardiography or nuclear medicine, and had an indication of cineangiography.

### Inclusion criteria

The study's project was described in line with the Declaration of Helsinki and approved by the Research Ethics Committee of the *Hospital Erasto Gaertner* (2274/13). All patients read, understood, and signed an informed consent form prepared according to Resolution 466/2012 of the National Health Council. The study included patients who presented ischemia on perfusion studies with pharmacological stress echocardiography or nuclear medicine due to severe obstructive lesions with > 50% obstruction in the left coronary trunk (LCT) and/or ≥ 70% in other segments, leading to ischemia in the region supplied by the affected artery.

### Exclusion criteria

We excluded from the study those cases with associated neoplasms, chronic obstructive pulmonary disease, renal insufficiency (creatinine > 2.0 mg/dL), hemorrhagic disease, acute myocardial infarction, stroke, or surgical treatment in the past 6 months, as well as coronary obstructions < 50% in the LCT territory and/or < 70% in other segments.

### Noninvasive functional evaluation methods

All patients included in the study underwent noninvasive functional evaluation with myocardial perfusion scintigraphy (MPS) and/or pharmacological stress echocardiography.

#### Myocardial perfusion scintigraphy

MPS was performed according to a standard protocol recommended by the American Society of Nuclear Cardiology (ASNC),^13^ both for the exercise and pharmacological stress (intravenous dipyridamole) protocols. The images were obtained with a tomographic gamma camera (Philips Cardio MD3), reconstructed with the program Cedars Quantitative Gated Spect, and interpreted by two independent investigators who concurred with the diagnosis of ischemia. The MPS images were qualitatively and quantitatively interpreted by more than one experienced investigator according to the ASNC recommendations. For the MPS quantification, we subjectively (visually) assigned a numerical value to each of the 17 segments in both phases, categorizing it as 0 (homogeneous uptake), 1 (slightly decreased uptake), 2 (moderately decreased uptake), 3 (markedly decreased uptake), or 4 (no uptake). The sum of the scores attributed to the 17 segments in the stress (SSS) and resting (SRS) phases allows a semiquantitative evaluation of the intensity and extent of the coronary disease.^13^

Exercise ECG was performed according to the Bruce protocol as per criteria established by the guideline of the Brazilian Society of Cardiology.^14^ Pharmacological stress was induced by intravenous injection of dipyridamole 0.84 mg/kg for 3 minutes, followed 4 minutes later by injection of the radiotracer (sestamibi-^99m^Tc) at a 555 to 740 MBq dose.^15^

The images were analyzed by two independent investigators and ischemia was considered to be present when both interpretations were in agreement.

#### Pharmacological stress echocardiography

The echocardiographic study with pharmacological stress was performed according to the criteria set by the guidelines of the Brazilian Society of Cardiology^13^ with continuous infusion of dobutamine at increasing doses every 2 minutes, starting with 5 µg/kg/min; when the maximal heart rate was not reached, atropine bolus was used at an initial dose of 0.25 mg.^16^

### Method of angiographic evaluation

All volunteers included in the study underwent coronary angiography. The coronary lesions diagnosed were initially classified according to their severity by quantitative coronary angiography (QCA). They were also assessed by ICUS for better quantification of the lesion areas. Additionally, the patients underwent FFR measurement and the results were compared with the ischemic areas suggested by noninvasive functional tests.

#### Quantitative coronary angiography

The angiographic images were evaluated by the main investigator (CRC) and the hemodynamic team of the *Hospital Cardiológico Costantini*. For that, we used a specific software to quantify obstructive coronary lesions (CASS version 5.7.4, Pie Medical Imaging B.V., The Netherlands).

In all cases, the images were obtained in different projections, always seeking a better visualization of the lesion and of the proximal and distal portions of the artery. Thus, it was possible to establish a mean reference diameter for the artery, the length of the lesion, the minimum luminal diameter, and the percentage of the diameter of the stenosis [(reference diameter - minimum luminal diameter)/(reference diameter x 100)] before and after the procedure. The calibration standard was established by the outer diameter of the catheter filled with contrast.^17^

#### Measurement of fractional flow reserve

To evaluate the impact of the lesion on the coronary flow, FFR was used according to established criteria,^18^ in which the distal pressure was measured with a 0.014-inche guide wire (Pressure Wire 4 Sensor, RADI Medical Systems, Uppsala, Sweden) or a Volcano Wave Wire (Volcano Inc., Rancho Cordova, California, USA) immediately distal to the stenosis, one at a time,^18^ during the period of maximal hyperemia induced by intravenous injection of adenosine 140 µg/kg/min through a large venous access in the antecubital vein. The aortic pressure was measured with a 6 or 7 F guide catheter. Lesions with a FFR ≤ 0.80 were considered to be responsible for the ischemia, as determined by the guidelines.^19^

#### Intracoronary ultrasound

The ICUS images were obtained with a rotating single element transducer with a 40 MHz frequency within a 2.6 Fr sheath and an automated transducer pullback with a speed of 0.5 mm/s, connected to an iLAB 2 scanner (Boston Scientific Corporation, Natick, USA) and Eagle Eye Platinum Intravenous Ultrasound (IVUS) Catheter (Volcano Corporation, San Diego, California, USA).

The images were digitized and analyzed according to the criteria of the Clinical Expert Consensus Document on Standards for Acquisition, Measurement and Reporting of Intravascular Ultrasound Studies (American College of Cardiology)^20^ and the program EchoPlaque 3.0.48 (INDEC Systems Inc., Mountain View, USA), respectively. Each millimeter of the arterial segments was analyzed with computerized planimetry to measure the lesion area and volume.^21^

### Study design

See figure 1 below.

**Figure 1:**
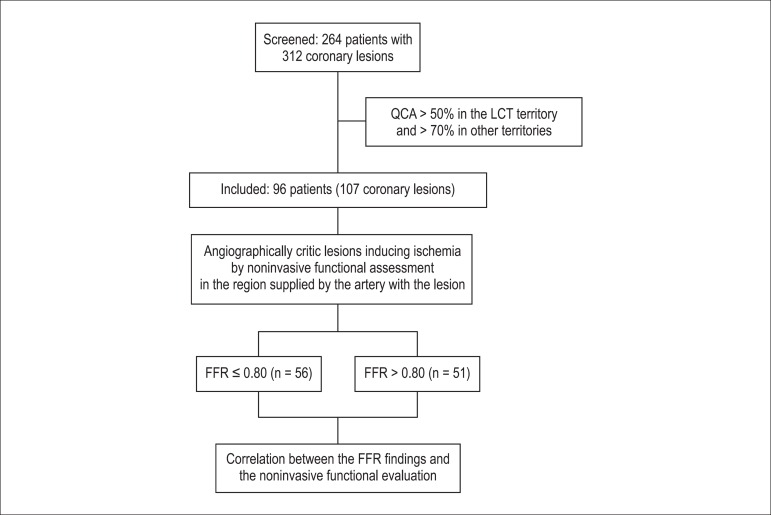
Study Design. QCA: quantitative coronary angiography; LCT: left coronary trunk; FFR: fractional flow reserve.

#### Statistical analysis

In the descriptive statistical analysis, the results of categorical variables are expressed as absolute frequencies and percentages. For continuous variables, we present mean ± standard deviation values. To verify homogeneity and normality, we applied the Levene and Shapiro-Wilk tests. To compare two groups in regard to quantitative variables, we used Student's *t* test for independent samples. When the comparison included more than two groups, we used one-way analysis of variance (ANOVA). Regarding categorical variables, the comparisons were performed using Fisher's exact test. To evaluate the cut-off values for quantitative variables associated with dichotomous outcomes of interest, we adjusted receiver operating characteristic (ROC) curves. Statistical significance was set at p values < 0.05. The data were analyzed with the programs IBM SPSS Statistics v.20 and GraphPad Prism v.6.05. We used logistic regression and ROC curve analysis to define the correlation coefficients between noninvasive and invasive functional evaluations with the FFR measurement.

## Results

In total, 107 obstructive lesions were diagnosed by angiography in the 96 patients included in the study. In 34% of the cases, the obstructions affected multiple vessels and in 81 cases (87% of the sample), the obstructions were categorized as type B/C according to the classification of the American College of Cardiology/American Heart Association.^22^ The anterior descending artery had the highest prevalence of lesions (52.34%).

Based on the assumption, grounded in the literature^19^ that coronary lesions with a FFR ≤ 0.80 should be deemed responsible for the myocardial ischemia, the following variables were compared between the FFR > 0.80 and ≤ 0.80 groups in the sample with ischemia detected by functional tests: modifiable and non-modifiable risk factors, clinical characteristics of the patients prior to the initiation of the clinical investigation, findings of noninvasive functional tests, and angiographic findings (QCA, ICUS, and FFR).

Table 1 presents the characteristics of the sample with regard to risk factors and clinical characteristics in the FFR > 0.80 and ≤ 0.80 groups. We observed similar results between both groups.

**Table 1:** Comparison of risk factors and clinical characteristics in the FFR ≤ 0.80 and FFR > 0.80 groups

**Clinical Characteristics**	**Total** **96 patients**	**FFR ≤ 0.8** **48 patients**	**FFR > 0.8** **48 patients**	p*
Age, mean ± SD	65.60 ± 10.34	65.8 ± 10.4	65.4 ± 10.4	0.90
Male gender, n (%)	66 (69)	31 (65)	35 (73)	0.46
Hypertension, n (%)	93 (97)	47 (98)	46 (96)	0.50
Obesity, n (%)	17 (18)	11 (23)	6 (12)	0.14
Diabetes mellitus, n (%)	48 (50)	23 (48)	25 (52)	0.41
Dyslipidemia, n (%)	93 (97)	46 (96)	47 (98)	0.50
Current smoking, n (%)	14 (15)	10 (21)	4 (8)	0.03
**Clinical Symptoms**	**Total** **96 patients**	**FFR ≤ 0.8** **48 patients**	**FFR > 0.8** **48 patients**	p*
Silent ischemia, n (%)	16 (17)	10 (21)	6 (13)	0.20
Stable angina, n (%)	40 (42)	20 (42)	20 (42)	0.09
Unstable angina, n (%)	33 (34)	13 (27)	20 (42)	0.09
Atypical angina, n (%)	6 (6)	4 (8)	2 (3)	0.33
Acute coronary syndrome, n (%)	1 (1)	1 (2)	0 (0)	0.50

(*) Fisher's exact test (categorical variables) or Student's t test for independent samples (quantitative variables); p < 0.05; n: number, SD: standard deviation.

**Figure 2:**
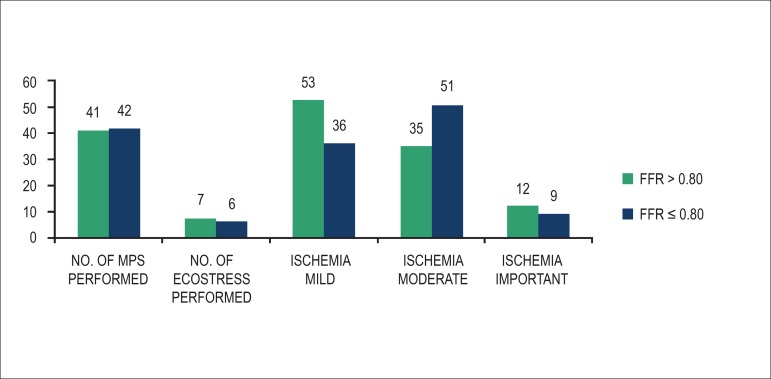
Percentage distribution of the functional tests performed. FFR: fractional flow reserve

Figure 2 presents the results of the noninvasive functional evaluations conducted in each group for the diagnosis of myocardial ischemia. In the FFR > 0.80 group, 41 patients (85%) underwent MPS, while seven (15%) underwent stress echocardiography. The corresponding numbers in the FFR ≤ 0.80 group were 42 (88%) and six (12%), respectively. Figure 2 also shows the results according to the classification of ischemia as mild, moderate, and important. We observed a higher frequency of mild ischemia in the FFR > 0.80 group and moderate ischemia in the FFR ≤ 0.80 group.

When we compared the groups in terms of angiographic characteristics, we observed a significant (p < 0.03) difference in regard to the anatomical location of the lesion, with a greater number of lesions in the anterior descending artery in the FFR ≤ 0.80 (Table 2).

**Table 2:** Comparison of angiographic characteristics in the general sample and in the FFR ≤ 0.80 and FFR > 0.80 groups

Angiographic characteristics	Total 107 lesions	FFR ≤ 0.8 56 lesions	FFR > 0.8 51 lesions	p*
Type B/C lesions, n (%)	87 (81)	42 (75)	39 (76.47)	0.07
Multivessel, n (%)	36 (34)	21 (37.5)	15 (29.41)	0.42
Bifurcation, n (%)	13 (12)	7 (12)	6 (11.76)	0.42
Left coronary trunk, n (%)	7 (6.54)	2 (3.57)	5 (9.80)	0.46
Left coronary trunk involving the proximal AD, n (%)	2 (1.87)	2 (3.57)	0 (0)	0.52
AD, n (%)	56 (52.34)	36 (64.29)	20 (39.21)	0.11
Diagonal, n (%)	5 (4.67)	3 (5.35)	2 (3.9)	0.65
Circumflex, n (%)	16 (14.95)	8 (14.28)	8 (15.68)	0.56
Circumflex marginal branch, n (%)	3 (2.8)	0 (0)	3 (5.88)	0.10
Right coronary, n (%)	15 (14.02)	4 (7.14)	11 (21.57)	0.05
Posterior descending - right coronary, n (%)	2 (1.87)	1 (1.78)	1 (1.97)	0.72
Saphenous vein graft, n (%)	1 (0.93)	0 (0)	1 (1.97)	0.47
QCA, RVD, mm (SD)	2.71 ± 0.63	2.70 ± 0.72	2.73 ± 0.53	0.31
QCA, stenosis diameter (%)	75.43 ± 6.68	75.5 ± 5.85	74.25 ± 8.5	0.39
QCA, length, mm (SD)	11.36 ± 5.19	12.12 ± 6.19	10.53 ± 3.71	0.11
**Ultrasonographic Characteristics**				
RVD, mm (SD)	2.99 ± 0.42	2.98 ± 0.40	3.15 ± 0.44	0.03
ICUS, stenosis diameter (%)	84.21 ± 8.46	84.25 ± 8.03	84.18 ± 9.00	0.96
ICUS, length, mm (SD)	19.89 ± 7.22	20.93 ± 8.02	18.76 ± 6.12	0.88
Fractional flow reserve (mean ± SD)	0.80 ± 0.10	0.72 ± 0.09	0.88 ± 0.04	0.00

(*) Fisher's exact test (categorical variables) or Student's t test for independent samples (quantitative variables); p < 0.05. AD: anterior descending; SD: standard deviation; RVD: reference vessel diameter; QCA: quantitative coronary angiography; ICUS: intracoronary ultrasound. * Considered statistically significant at p < 0.05.

Table 2 also shows that when the QCA was compared with respect to the diameter of the stenosis, there was no significant difference between lesions with FFR ≤ or > 0.80 (74.25 ± 7.2% *versus* 75.5 ± 6.84%, respectively). Also, no significant differences were observed when the length of the lesion was compared between the FFR ≤ 0.80 and > 0.80 groups: 12.12 ± 5.22 mm *versus* 10.53 ± 4.24 mm, respectively, on QCA evaluation and 20.92 ± 7.27 mm *versus* 18.76 ± 7.22 mm, respectively, on ICUS evaluation.

Table 3 shows the characteristics of the predictors of ischemia for a FFR ≤ 0.80. Considering the sensitivity, specificity, and positive and negative predictive values, we found a reference arterial diameter of < 2.62 mm, and minimal luminal diameters of < 0.36 mm on QCA and < 2.50 mm on ICUS.

**Table 3:** Characteristics of the analysis of ischemia predictors for a FFR ≤ 0.80

Variable	AUC (%)	95% CI	Accuracy	Values associated with a FFR ≤ 0.80 (cut-off values)	Sensitivity (%)	Specificity (%)	PPV (%)	NPV (%)
QCA diameter	0.5	0.39 - 0.62	53.3%	≥ 76%	48.2	58.8	56.3	50.8
ICUS diameter	0.49	0.38 - 0.60	52.3%	≥ 86%	57.1	47.1	54.2	50.0
QCA RVD (mm)	0.54	0.43 - 0.65	57.0%	< 2.62	57.1	56.9	59.3	54.7
QCA MLD (mm)	0.53	0.42 - 0.64	57.0%	< 0.36	48.2	66.7	61.4	54.0
ICUS MLD (mm)	0.54	0.43 - 0.65	57.9%	< 2.50	53.6	62.7	61.2	55.2
QCA LL (mm)	0.59	0.48 - 0.70	64.5%	≥ 9.68	66.1	62.7	66.1	62.7
ICUS LL (mm)	0.58	0.47 - 0.69	57.9%	≥ 20	51.8	64.7	61.7	55.0

QCA: quantitative coronary angiography; ICUS: intracoronary ultrasound; RVD: reference vessel diameter; MLD: minimal luminal diameter; LL: lesion length; PPV: positive predictive value; NPV: negative predictive value; AUC: area under the ROC curve; 95% CI: 95% confidence interval for the AUC. For these calculations, the prevalence of FFR ≤ 0.80 in this study population was estimated from the sample results (56/107 = 52.3%).

## Discussion

The main findings of this study were: 1) in the overall evaluation of the sample, the descending anterior artery showed the highest prevalence of lesions (52.34%), while 87% of the sample presented type B/C obstructions; 2) when patients with ischemia diagnosed by a noninvasive functional test were divided into FFR > 0.80 and ≤ 0.80 groups, there were no significant differences between both groups in regard to modifiable and non-modifiable risk factors, as well as clinical symptoms leading to the investigation. In the angiographic data evaluated, there was a significant difference with respect to the anatomical location of the lesion, with more common lesions in the left anterior descending artery in the FFR ≤ 0.80 group; 3) correlation analysis for FFR ≤ 0.80 considering the sensitivity, specificity, positive and negative predictive values, accuracy and ROC curve relative to the presence of ischemia and stenosis degree and length did not show values with significance or strong correlation.

For some authors, the cut-off value of 0.80 for the FFR may represent more than an anatomic evaluation. Pijls et al.^22^ studied 45 patients with angiographically questionable stenoses according to their angiographic severity. In 24 and 21 patients with 44 ± 9% and 41 ± 8% percent stenoses, respectively, their results suggested that the FFR had a greater accuracy to distinguish stenoses with a potential hemodynamic impact (sensitivity of 88% and specificity of 100%) compared with exercise testing, MPS, and stress echocardiography.

Other studies have been published using the FFR as a measurement to recommend or not recommend PCI, including the DEFER study,^11^ which evaluated 325 patients divided into three groups, none of whom had undergone functional evaluation to justify the procedure. The patients were randomized to group 1 (defer; immediate PCI or not, n = 91, no prior functional tests and FFR ≥ 0.75, undergoing optimized clinical treatment), group 2 (reference; n = 144, no prior functional tests and FFR < 0.75, undergoing immediate PCI), and group 3 (perform; n = 90, no prior functional tests, with FFR ≥ 0.75 and mean stenosis percentage of 48 ± 10%, undergoing, nonetheless, immediate PCI). The 5-year follow-up in the DEFER study^23^ showed consistent results, with a risk of death or infarction of 1% per year in the population whose treatment was deferred based on the FFR. It is worth noting that the patients in the *perform* group who had no clinical or noninvasive functional criteria for PCI presented a 7.9% rate of death/acute myocardial infarction at 5 years. However, it is unclear whether these results would be similar had noninvasive diagnostic tests such as MPS been performed. In the present study, unlike the methodology of the DEFER study, patients undergoing coronary angiography had a positive functional assessment of myocardial ischemia and, as a result, we noted that there was no significant or strong correlation (sensitivity/specificity), positive/negative predictive values, and accuracy in relation to the degree or extension of the stenosis and presence of ischemia. Although the FAME study^19^ showed that 60% of the patients had obstructive lesions > 70% and nearly 20% had lesions > 90%, these patients had not undergone noninvasive functional tests that could be confronted with the values obtained by FFR measurement.

It is clear that the decision of coronary intervention should be based on objective evidence of the functional and anatomical impact of the coronary narrowing;^24,25^ this evidence helps to stratify the disease risk and future coronary events, providing better guidance in terms of therapeutic approach.^26,27^ Patients with significant areas of ischemia have a worse prognosis when maintained on clinical treatment.^28^ If the ischemia negatively affects the individual's daily life due to the occurrence of symptoms, revascularization may bring major benefits, as shown in the COURAGE study, which demonstrated better symptom control with revascularization;^29^ even asymptomatic patients with moderate/important ischemia show better outcomes in terms of reduction of adverse events after revascularization of the lesion.^30^

A very important issue that should be addressed in this discussion is related to the numerous changes that the methodology used for FFR measurement has undergone during the evolution of interventional cardiology. These changes relate to:

- The ideal dose of adenosine: Pijls et al.^22^ have validated the method using an intravenous infusion of adenosine at a dose of 140 µg/kg/min to induce maximal hyperemia. The DEFER study^11^ used two methods for adenosine administration: intravenous, at a dose of 140 µg/kg/min, and intracoronary, at a dose of 15 µg in the right coronary and 20 µg in left coronary. The ISCHEMIA study,^31^ in turn, proposed that the dose of 140 µg/kg/min should be doubled when the FFR results are ≥ 0.81 or ≤ 0.82. In addition, De Luca et al.^32^ showed that intracoronary adenosine at increasing doses of up to 720 µg progressively decreased the FFR values. We should also emphasize that the infusion of adenosine at a dose of 140 µg/kg/min may not produce absolute maximal vasodilation in the subepicardial infarction in all patients.^33^
- Route of administration: different protocols suggest different administration routes, including intravenous, intracoronary, and central lines.
- Time to maximal hyperemia: In 2013, Tarkin et al.^34^ published a study showing that the measurements should only be obtained when steady-state hyperemia has been reached for ≥ 60 seconds during continuous intravenous infusion of adenosine, which is not consistent with protocols used in previous studies.^12^
- Ideal cut-off value: The cut-off value to detect ischemia with a sensitivity of 90% and specificity of 100% is 0.75. Values below 0.75 are almost always associated with myocardial ischemia, while stenosis associated with FFR greater than 0.80 are almost never associated with ischemia, creating a gray area for FFR values between 0.75 and 0.80.^35^ To increase to close to 100% the sensitivity to detect ischemia, a FFR cut-off value of 0.80 has been recently used.^12^ In a recent study, Petraco et al.^36^ suggested that the gray zone for the FFR measurement is between 0.75 and 0.85. In clinical practice, this means that each time a single FFR measurement falls between 0.75 and 0.85, there is a chance that a recommendation for revascularization guided by FFR may change if the measurement is repeated after 10 minutes; the chance becomes greater as the FFR result becomes closer to 0.80. Based on the classic flow dynamics equation, in which the resistance to the flow across the stenosis is dependent on both the length and diameter of the stenosis, Lopez-Lopez-Palop et al.^37,38^ and Jaffe et al.,^39^ recently showed that the length of the lesion is more important than its diameter when the functional impact of the lesion is estimated. It is important to emphasize that in our registry, the longer was the lesion, the greater was the correlation with the positive FFR, corroborating the theory defended by these authors.

It is questionable if the 0.80 cut-off value for the FFR measurement is ideal to quantify lesions and whether it is really possible to define a patient's therapy based on this method alone since this study was unable to show reproducibility in severe lesions with noninvasive functional tests to confirm its physiological meaning.

Based on the findings of this study and this sample, we believe that it is precocious to adopt the cut-off value of 0.80 for FFR measurement as a gold standard with a class of recommendation I and level of evidence A^40^ in defining the treatment strategy for coronary artery disease. Some barriers still need to be overcome, such as the definition of the actual value of the ideal reference for the cut-off measurement, the time to hyperemia, and the dose and ideal administration route for FFR measurement.

### Study limitations

The number of patients included in the study was low. A continuity of the study including a greater number of participants is suggested.

## Conclusion

This study found no correlation between FFR values (cut-off value of 0.80) with the presence of myocardial ischemia obtained by noninvasive functional studies in angiographically severe coronary lesions assessed by QCA.
